# Prevalence of flu-like syndrome in healthcare workers in Brazil: a national study, 2020

**DOI:** 10.11606/s1518-8787.2023057004384

**Published:** 2023-03-15

**Authors:** Ada Ávila Assunção, Ricardo da Silva Freguglia, Marcel de Toledo Vieira, Larissa da Silva Marioni

**Affiliations:** I Universidade Federal de Minas Gerais Faculdade de Medicina Departamento de Medicina Preventiva e Social Belo Horizonte MG Brasil Universidade Federal de Minas Gerais. Faculdade de Medicina. Departamento de Medicina Preventiva e Social. Belo Horizonte, MG, Brasil; II Universidade Federal de Juiz de Fora Faculdade de Economia Departamento de Economia Juiz de Fora MG Brasil Universidade Federal de Juiz de Fora. Faculdade de Economia. Departamento de Economia. Juiz de Fora, MG, Brasil; III Universidade Federal de Juiz de Fora Instituto de Ciências Exatas Departamento de Estatística Juiz de Fora MG Brasil Universidade Federal de Juiz de Fora. Instituto de Ciências Exatas. Departamento de Estatística. Juiz de Fora, MG, Brasil; IV National Institute of Economic and Social Research London United Kingdom National Institute of Economic and Social Research. London, United Kingdom

**Keywords:** Patient Care Team, Signs and Symptoms, Respiratory, Risk Factors, Socioeconomic Factors, Health Surveys, Pnad Covid-19

## Abstract

**OBJECTIVE:**

To evaluate the prevalence of reported symptoms of flu-like syndrome (FS) among HCW and compare HCW and non-HCW on the chance of reporting these symptoms, this study analyzed data of a population-based survey conducted in Brazil.

**METHODS:**

A cross-sectional analysis was performed with self-reported data from the *Brazilian National Household Sample Survey* (PNAD Covid-19) from May 2020. The authors analyzed a probability sample of 125,179 workers, aged 18 to 65, with monthly income lower than US$ 3 500. The variable HCW or non-HCW was the covariate of interest and having reported FS symptoms or not was the outcome variable. Authors tested interactions of HCW with other covariates. A logit model – when controlling for sociodemographic, employment, and geographic characteristics – investigated the chance of HCW reporting FS compared to non-HCW.

**RESULTS:**

HCW have a significant effect (odds ratio of 1.369) on reporting FS symptoms when compared to non-HCW. HCW account for 4.17% of the sample, with a higher frequency of FS (3.38%) than observed for non-HCW (2.43%). Female, non-white and older individuals had higher chance to report FS.

**CONCLUSIONS:**

The HCW had a higher chance of reporting symptoms than non-HCW aged over 18 years in the labor force. These results emphasize guidelines for preventive measures to reduce workplace exposures in the healthcare facilities. The prevalence is disproportionately affecting HCW women and HCW non-whites. In the regions North and Northeast the steeper progression is consistent with the hypothesis of socioeconomic factors, and it explains the greater prevalence in HCW and non-HCW living in those territories.

## INTRODUCTION

Coronavirus disease 2019 (Covid-19) pandemic has impacted on societies and infrastructure of healthcare services. The first confirmed case in Brazil was reported on February 26, 2020. Compared to other countries, in November 2021, Brazil ranked third in number of accumulated cases, and second in number of deaths due to this infection^1^. Healthcare workers (HCW) are deemed as one of the groups with the highest risk of exposure to Covid-19^2 , 3^. In the systematic review and meta-analysis across the 28 studies, 51.7% (95%CI: 34.7–68.2) prevalence of testing positive from the reports of HCW at the frontline^4^.

There is scarce data on occurrence of Covid-19 in HCW in Brazil^5^. Nonetheless, it is known that diagnosis has been confirmed in 23.8% of cases of flu-like syndrome (FS) reported up to November 2021 for these workers. The health professions with the highest records of confirmed cases were nurse technicians/licensed practical nurses, followed by registered nurses and physicians^1^. However, these official data are not disaggregated, and underreporting and low testing problems have been identified^4 , 5^.

Understanding the prevalence of the characteristic symptoms of Covid-19 in HCW is justified by at least six reasons. First, the Brazilian protocol considers FS a suspected case of Covid-19^6 , 7^. Second, HCW are crucial to face the pandemic^2^. Third, if the results on psychosocial suffering related to healthcare facilities^8^ – high prevalence of suspected cases and deaths in this professional category^5^ – were published in Brazil, the impropriety of working conditions in health services would have been documented and associated to its factors^9^. One out of four HCW (26%) in Brazil reported lack of information to prevent the infection. Furthermore, one out of five complained about shortage of personal protection equipment^10^. Some examples in the same line demand monitoring and changes in the working environment^11^. Fourth, cross-transmission is common; in other words, HCW contaminated during patient care is a source of risk to their colleagues and other patients. Additionally, the HCW are a source of risk to their families and communities in general since they work outside home^3^. Fifth, surveillance systems and health promotion for HCW lack information on characteristics of symptomatic individuals^12^. Lastly, assessing the characteristics of symptomatic HCW at their homes is fundamental to understand the behavior of the disease in this occupational category. Therefore, it allows comprehending about the distribution of symptoms in the country and not only at workplaces, which is supported by occupational health services or data collection.

In order to evaluate the prevalence of reported symptoms of FS among HCW and compare HCW and non-HCW on the chance of reporting these symptoms, this study analyzed data of a population-based survey conducted in Brazil.

## METHODS

Between May and November 2020, the Brazilian Institute of Geography and Statistics (IBGE), in partnership with the Brazilian Ministry of Health, conducted a household-based probability sample and longitudinal survey. Applying telephone interviews to collect data, they estimated the number of people with FS and the impact of Covid-19 pandemic on the Brazilian labor market. This effort is entitled Brazilian National Household Sample Survey on Covid-19 (PNAD Covid-19, acronym in Portuguese)^13^.

### Study Design and Participants

Data collection started in May 2020 in approximately 48 thousand households per week and in 3,364 municipalities throughout all states of the country - approximately 193 thousand households per month. The sample size defined by IBGE is large enough to guarantee statistical conclusions for domains, usually considered by the traditional Continuous PNAD^14^.

Selected in two stages with stratification of primary sampling units (PSUs), in the first stage of the sample, they were chosen with a probability proportional to the number of households within each defined stratum. In the second stage, 14 permanent private households were selected within each PSU^14^.

As PNAD Covid-19 collected data that was later publicly available on the Internet and without any form of individual identification, there was no need for the study to be submitted to a Research Ethics Committee with Human Beings.

### Data and Descriptive Statistics

For the empirical analysis, we kept only individuals aged 18 to 65 years, who were in the labor market. Moreover, the study excluded individuals who earned more than US$ 3,500 per month - 2.87% of original sample - to take out outliers from the sample. Overall, we analyzed data on 125,179 individuals.

Our explanatory variable of interest is a dummy to determine if a worker is a HCW. In order to construct this variable, we used the following question from the PNAD Covid-19 questionnaire: What kind of job, position or function do you have in your work (single or main)? Physicians, nurses, healthcare professionals with a university degree or technicians, and healthcare professionals with further education were considered HCW, and other professionals non-HCW. The HCW variable equals to one if a person was classified as HCW; otherwise, the variable was zero.

Therefore, the outcome variable considered FS the most common manifestation of Covid-19^6 , 7^. Hence, a case of FS symptoms (same as suspected case of Covid-19) is defined when the respondent reported fever, anosmia or ageusia; or cough, fever and difficulty breathing; or fever, cough, and chest pain. A dichotomous variable is equal to one if a person had FS symptoms; otherwise, the variable was zero. The symptoms period refers to the week before the subject’s interview. Moreover, the variable formal job was defined based on IBGE’s criterion, i.e., informal workers are: private sector employers with no formal contract; domestic workers with no formal contract; employers who do not contribute to the social security; self-employed workers who do not contribute to the social security; or unpaid workers assisting a householder or relative. A dummy variable is equal to one if a worker did not fit into any of the categories, that is, formal workers; otherwise, it was equal to zero.

We estimated the relative frequencies of FS considering individual characteristics (sex, age, and education levels), employment characteristics (type and number of jobs) and regional information (dummies of Brazilian regions).

The prevalence rate of FS is the number of cases per 100 thousand individuals in subgroup i, group j:


$$FS_{ij}={\mathrm{Cases}}_{ij}\times 100,000/{\text{ Population }}_{ij}$$

where *i* are demographic and employment characteristics, and *j* is either HCW or non-HCW.

### Statistical Analysis

As described in the previous section, in this article the outcome variable *y* is a dummy that takes the value one if the individual reports FS symptoms; otherwise, the value is zero. We used a multiple Logit model^15 , 16^ to investigate the prevalence of healthcare workers compared with non-healthcare workers, which reported FS compatible symptoms. Point and variance estimation are performed to allow weights and sampling design features of PNAD Covid-19 for the survey through Stata Version 15 svyset of commands (https://www.stata.com/manuals15/svysvyset.pdf).

In order to analyze the prevalence of FS cases by occupation (HCW and non-HCW), and examine whether other characteristics – such as individual, job, and geographical features – are associated to FS symptoms, we estimated odd ratios (ORs)^17^ with their respective 95%CI, following equation 6:


$$P\left(FS_{i}\right)=\Phi \left(\beta_{0}+\beta_{1}HCW_{i}+\beta_{2}X_{i}\right)+u_{i}$$ (6)

Whereupon, *FS*_*i*_ is an indicator equal to 1 if worker *i* reported having influenza-like illness symptoms (and 0 otherwise); *Φ* (⋅) is the standard logistic cumulative distribution function (CDF); *HCW* is a dummy variable if one works as a healthcare worker; *X*_*i*_ *is* a vector with individual data for a set of variables (sex, age, and education levels), job (type and number of jobs), and geographical characteristics (dummies of Brazilian regions); *β*_1_ and *β*_2_ are the vectors of coefficients to be estimated, and *u*_*i*_ is the error term.

## RESULTS

HCW account for 4.17% of the sample (N-hat = 2,899,470) ( Table 1 ). In this group, the prevalence of FS symptoms reporting was higher (3.38%) than that observed in the non-HCW group (2.43%). Education levels and income were higher in the first group as compared with the second. Regarding the sex, females were 76.42% in the HCW group while 43.61% in the non-HCW group. There were fewer non-white individuals in the HCW group (46.43%) comparing with the non-HCW group (53.67%). Distribution per age and geographic region of residence were similar in both groups. Informal work was less frequent in the HCW group (12.53%) than in the non-HCW group (33.65%). Multiple job holding was reported by 17.80% of HCW *versus* 7.85% of non-HCW. Most public employment contracts were observed in the HCW group (46.21%), contrasting with more private employment contracts (47.50%) and self-employed workers (30.03%) in the non-HCW group. Unpaid service delivered to families and house chores were informed only in the non-HCW group.

**Table 1:** Demographic and employment characteristics of symptomatic HCW ≥ 18 years (N-hat = 2,899,470) and non-HCW adults ≥ 18 years (N-hat = 66,667,290), Brazil, 2020.

Variable	HCW (Healthcare workers)		Non-HCW (Non-healthcare workers)	
	
	Mean/proportion	Standard error	Mean/proportion	Standard error
FS symptoms^a^				
Yes	3.38	-	2.43	-
No	96.38	-	97.57	-
Age	40.05	0.2038	40.02	0.0489
Wage (R$)	3,600.45	78.3495	2,141.90	16.8695
Sex				
Men	23.58	-	56.39	-
Women	76.42	-	43.61	-
Ethnic group				
White	53.57	-	46.33	-
Non-white	46.43	-	53.67	-
Education				
Illiterate	-	-	1.21	-
Incomplete primary education	-	-	17.97	-
Complete primary education	-	-	7.72	-
Incomplete secondary education	-	-	7.96	-
Complete secondary education	33.27	-	34.64	-
Incomplete tertiary education	6.58	-	7.63	-
Complete tertiary education	46.07	-	17.81	-
Postgrad, masters or doctorate	14.07	-	5.06	-
Region				
North (N)	6.07	-	7.13	-
Northeast (NE)	25.75	-	22.32	-
Midwest (CO)	7.84	-	8.19	-
Southeast (SE)	45.49	-	45.39	-
South (S)	14.83	-	16.96	-
More than one job				
Yes	17.80	-	7·85	-
No	82.20	-	92.15	-
Formal job contract				
Yes	87.47	-	66.34	-
No	12.53	-	33.65	-
Occupation				
Public sector	46.21	-	13.16	-
Private sector	38.26	-	47.50	-
Self-employed	12.76	-	30.03	-
Employer	2.77	-	3.23	-
Domestic worker	-	-	6.06	-
Unpaid family service worker	-	-	0.03	-
n	4,992		120,187	
N-hat	2,899,470		66,667,290	

HCW: healthcarer workers.

^a^ For the purposes of this study, a case of FS (flu-like syndrome) is defined when the respondent reported:

(i) a fever, anosmia or ageusia or cough, and difficulty breathing or (ii) fever, cough and chest pain.

Source: PNAD COVID-19 data, own calculations.

Among women, the prevalence of FS was 21% higher in the HCW group compared to non-HCW ( Table 1 ). Concerning men, the prevalence was 68% higher in HCW when compared to the non-HCW group. As for the distribution by age group, the highest prevalence of FS was observed among HCW aged 18 to 29, 30 to 39 and 50 to 59 years. Emphasis on the first and third age groups, whose prevalence of FS are 93% and 67% higher in the HCW group compared to the non-HCW group. In the fourth or last age group (60 years and over), the prevalence of FS is 232% higher in the non-HCW group.

Regarding non-whites, the frequency was 33.4% higher in the HCW group when compared to non-HCW. The prevalence is lower for higher levels of education in both HCW and non-HCW ( Table 1 ).

In the group of informal workers, FS frequency is 21% lower in the HCW group when compared with non-HCW Table 1 . The result is inverted for those with formal employment, as the prevalence of FS in HCW is 54% higher compared to non-HCW. Among the non-HCW group, the prevalence in the public sector is 13%, while for the HCW it is 46%. Concerning workers with more than one job, the number of HCW with FS is 49% higher than the amount of non-HCW ( Table 1 ).

**Figure 1:**
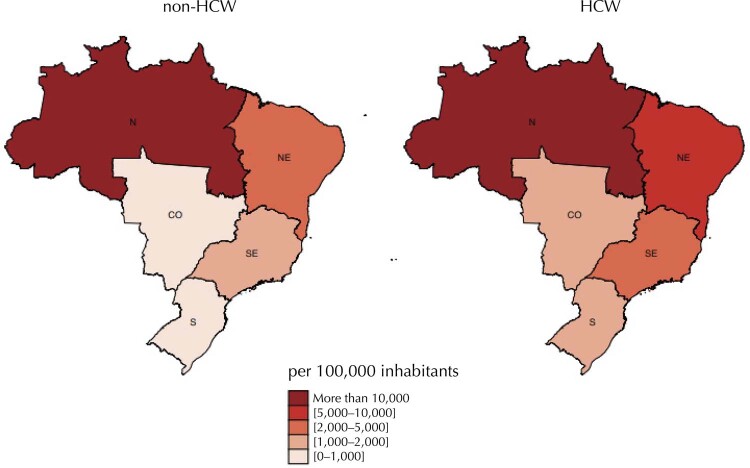
Prevalence of reported symptoms of Covid-19 in healthcare workers (HCW) adults ≥ 18 years and non HCW adults ≥ 18 years among Brazilian regions, 2020.

Figure 1 shows a map that uses a color scale according to the prevalence of SF among Brazilian regions. Warmer colours (redder) indicate the regions with a higher prevalence of FS, while cooler colours (whiter) indicate the regions with a lower prevalence of FS. Comparing HCW with non-HCW, the prevalence of FS is higher in the Northeast, Southeast, South and Midwest regions - except the North region, where this number is the same for both groups.

**Figure 2:**
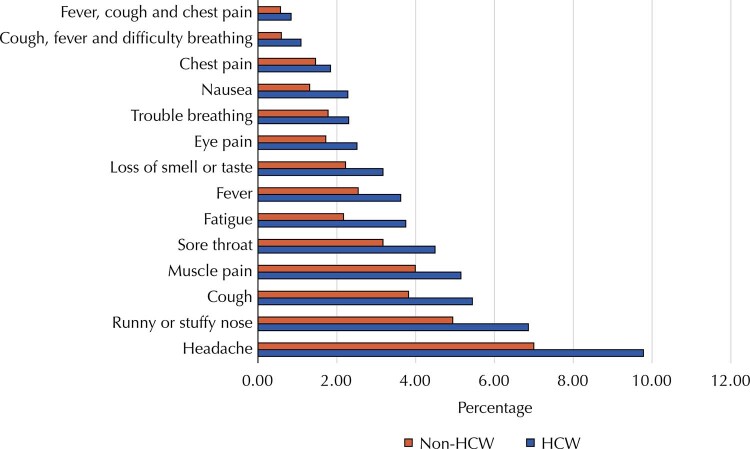
Prevalence of reported symptoms of Covid-19 in healthcare workers (HCW) adults ≥ 18 years and non HCW adults ≥ 18 years, Brazil, 2020.

HCW group reported a higher symptoms frequency ( Figure 2 ) for each of them analyzed separately, especially headache, runny or stuffy nose, cough, fatigue (9.78%, 6.86%, 5.44% and 3.75%, respectively) compared with that reported by non-HCWs (7.00%, 4.94%, 3.82% and 2.17%).

Table 2 displays the odds ratios of Logit estimates for equation (6). We estimated all the regressions using the PNAD Covid-19 survey weights and allowing design features for the sampling. As previously defined, our outcome is an indicator variable that equals 1 if worker *i* reported having influenza-like illness symptoms (and 0 otherwise). Considering cases of FS as suspected Covid-19, we expected a higher chance of infection among the HCW, when compared with adults in other occupations or unemployed. According to our preferred model, after controlling for sociodemographic, employment, and geographic characteristics, HCW have a positive and significant effect, since this group is 36.9% more likely to report FS symptoms than non-HCW.

**Table 2:** Logit odds ratio of HCW on flu-like syndrome (FS).

Independent variables	(1)			(2)			(3)			(4)		
			
	OR	95%CI	p	OR	95%CI	p	OR	95%CI	p	OR	95%CI	p
Healthcare workers (HCW)	1.369***	**1.12–1.68**	**< 0.002**	1.770***	**1.19–2.62**	**< 0.005**	1.608***	**1.17–2.21**	**< 0.003**	2.173*	**0.97–4.85**	**< 0.058**
	**(0.142)**			**(0.356)**			**(0.261)**			**(0.890)**		
Interaction HCW*Female				0.713	0.46–1.11	< 0.136						
				(0.162)								
Interaction HCW*Non-white							0.768	0.51–1.15	< 0.202			
							(0.159)					
Interaction HCW*Age										0.988	0.97–1.01	< 0.243
										(0.009)		
Individual characteristics	Yes			Yes			Yes			Yes		
												
Job characteristics	Yes			Yes			Yes			Yes		
												
Regional characteristics	Yes			Yes			Yes			Yes		
												
Observations	125,179			125,179			125,179			125,179		

Source: PNAD Covid-19 data, own calculations.

Notes:

1. The odds ratio means the probability of an event to occur in a given group.

2. Linearized standard erros in parentheses.

3. Strata with single sampling unit centered at overall mean.

4. OR: odds ratio; 95%CI: 95% confidence interval.

5. For the purposes of this study, a case of FS (flu-like syndrome) is defined when the respondent reported: (i) a fever, anosmia or ageusia or cough, and difficulty breathing or (ii) fever, cough and chest pain.

6. Standard errors in parentheses; *** p < 0.01, ** p < 0.05, * p < 0.1.

Presented in columns 2, 3, and 4 in Table 2 , we performed additional estimations to obtain robust results. Even when considering the interactions of HCW with sex, racial group, and age, our results show a higher chance of HCW to report FS symptoms than non-HCW. Depending on the choice of interaction, HCW are estimated to be 77.0%, 60.8%, and 117.3% more likely to report FS symptoms than non-HCW.

## DISCUSSION

The study provided a snapshot of the situation for HCW during the first epidemiologic weeks of the pandemic in Brazil. To the best of our knowledge, PNAD Covid-19 is the original population-based study to evaluate the prevalence of reports of FS symptoms among adults in the labor force.

Regardless of sex, race, and age, the HCW had a higher chance of reporting symptoms than non-HCW. When testing the interaction of health-related occupations with other variables this result - corroborated by the literature^1^remained, suggesting that the effect of occupational exposure in healthcare facilities prevailed over other keys of infection frequency in HCW. However, the prevalence is disproportionately affecting HCW women and HCW non-whites.

At the time of study (May, 2020), Brazil ranked second among countries in speed of transmission^19^. When the services were not adequately prepared to respond to this impact, the exponential increase in volume of care delivered, put pressure on HCW^20^. The existence of a gap of protection for HCW probably explains the higher chance of reporting symptoms in this group, as compared to non-HCW^6^. Protection measures were not developed immediately, given the technical unpreparedness in the face of an unknown infection. In China, approximately 90% of HCW infected up to February 24, 2020, were from the province of Hubei, where the outbreak started in the country^18^.

In Brazil, eighty days after the first death, the epidemic curve was faster in the regions North and Northeast. The steeper progression in these states is consistent with the hypothesis of socioeconomic factors, and it explains the greater prevalence in HCW and non-HCW living in those territories^21^. Poorer living conditions increase the chance of exposure to the viral material, since in this situation the individuals, HCW and non-HCW are more likely to use public transportation, live in crowded households, and circulate in neighborhoods with poor sanitation.

Critical situations, such as the Covid-19 pandemic, revealed effects of the gender inequality^22^. This occupational group comprises mostly women, who are involved in direct care of patients, working at bedside and in first emergency procedures, placing them at increased risk of viral infection^20^. One tenth of hospital HCW diagnosed as acute SARS-CoV-2 infection, and half of them were female nursing staff^3^. In Brazil, the majority of community health workers are females, who deliver care to citizens in their respective houses^5^.

The greater susceptibility in older individuals was well documented in the first months of the pandemic^23^. How could we explain the greater chance of reporting symptoms in younger HCW? First, HCW who carry out their tasks close to patients are in the active phase of life; therefore, the hypothesis about different age-related occupational exposure is plausible. Second, the outcome analyzed in the study is reports of symptoms, hence diverse from the results of greater vulnerability of older adults in the confirmed cases of Covid-19.

The lesser chance of reporting symptoms in non-HCW with higher education levels was expected, for this reason educational experience fosters development of skills to face hardship. In such situation, the individuals adopt self-protection measures faster, because they are more sensitive to messages about prevention. Furthermore, education level is associated to better general health status^24^.

Confirming previous results on racial health disparities^25^, the chance of reporting symptoms was greater among non-white respondents as compared to whites. In Brazil, the income of white individuals in 2018 was 73.9% higher than that of non-whites; 17.9% of non-white individuals *versus* 11.5% of whites had no piped water supply in 2018; and 42.8% of non-whites *versus* 26.5% of whites lived in areas with no sewer system^26^. These socioeconomic conditions are determinants of health status and can be added up to general housing conditions, which are also unfavorable for non-whites, thus interfering in measures to prevent and control FS and Covid-19.

Among reported symptoms, headache stood out and it was also the most frequent symptom in Sweden^27^. In the United States, 65% of positive Covid-19 HCW presented headache in the initial phase of the infection^28^.

Interpretation of results has many limitations. As the PNAD Covid-19, at least three types of bias are probable in research based on self-reporting: namely memory, verification, and information. Since the questions were about a recent period, the first bias may have been minimized two months after the first case of confirmed infection in Brazil. Regarding verification, a selection bias is expected in countries with inappropriate telephone coverage. Nonetheless, the rate of households with landline has recently expanded to 78.5% in Brazil^29^.

Diverse modalities of data collection may lead to different results, even considering the coincident format and content of questions. This mode effect is produced, among other factors, by interference of respondents judging the social acceptance of their answer, possibly stronger in face-to-face interviews than over the phone^30^. Communication problems are more probable in this mode since they depend on quality of the telephone network system. Interviewees are less tolerant to duration of the interview over the phone. Furthermore, the chance of errors is greater, as well as of interviewees giving up, as compared to the face-to-face mode^31^. Taking this limitation into account, data collection by interview over the phone, using a computer-assisted questionnaire, had many advantages regarding costs and speed of data processing^32^. Since commuting was not necessary, access to participants from remote areas was possible, and the interviewer did not have no move through unsafe areas. This modality has already been traditional in health-related surveys in Brazil, and its consistency of results has already been proven^33^_. _

PNAD Covid-19 is a robust investigation, because it took advantage of the established capacity of IBGE to obtain a representative sample for all states of the country. This survey avoided the inclusion bias, that is, it addressed a contingent of cases with less typical clinical picture, including the oligosymptomatic and those who had no access to health services. Finally, the questions enabled the researchers to combine reports of symptoms to consistently define cases^32^.

The primary objective of public health is to prevent dissemination of diseases. In order to achieve this goal, the results presented point to the need for interrelating sectoral policies, to identify who are the symptomatic workers, how many there are, and in which healthcare facilities they work. It is suggested to reinforce contingence plans to monitor these workers. Administrative and engineering measures, such as access to hand washing stations, hygiene practices, and cleaning of the working space, availability of personal protective equipment, and other factors, probably vary in terms of quality and quantity among the occupational subgroups and different healthcare facilities. All these plans justify implementing worker health surveillance systems coordinated with epidemiologic surveillance systems^5^.

## CONCLUSION

Our results highlight the HCW had a higher chance of reporting symptoms than non-HCW aged over 18 years in the labor force. These results emphasize guidelines for preventive measures to reduce workplace exposures in healthcare facilities.
